# Context-dependent switch in chemo/mechanotransduction via multilevel crosstalk among cytoskeleton-regulated MRTF and TAZ and TGFβ-regulated Smad3

**DOI:** 10.1038/ncomms11642

**Published:** 2016-05-18

**Authors:** Pam Speight, Michael Kofler, Katalin Szászi, András Kapus

**Affiliations:** 1 Keenan Research Centre for Biomedical Science of St Michael's Hospital, University of Toronto, Toronto, Ontario, Canada M5B 1T8; 2 Department Surgery, University of Toronto, Toronto, Ontario, Canada M5P 1T5; 3 Department Biochemistry, University of Toronto, Toronto, Ontario, Canada M5S 1A8

## Abstract

Myocardin-related transcription factor (MRTF) and TAZ are major mechanosensitive transcriptional co-activators that link cytoskeleton organization to gene expression. Despite many similarities in their regulation, their physical and/or functional interactions are unknown. Here we show that MRTF and TAZ associate partly through a WW domain-dependent mechanism, and exhibit multilevel crosstalk affecting each other's expression, transport and transcriptional activity. Specifically, MRTF is essential for TAZ expression; TAZ and MRTF inhibit each other's cytosolic mobility and stimulus-induced nuclear accumulation; they antagonize each other's stimulatory effect on the α-smooth muscle actin (SMA) promoter, which harbours nearby cis-elements for both, but synergize on isolated TEAD-elements. Importantly, TAZ confers Smad3 sensitivity to the SMA promoter. Thus, TAZ is a context-dependent switch during mechanical versus mechano/chemical signalling, which inhibits stretch-induced but is indispensable for stretch+TGFβ-induced SMA expression. Crosstalk between these cytoskeleton-regulated factors seems critical for fine-tuning mechanical and mechanochemical transcriptional programmes underlying myofibroblast transition, wound healing and fibrogenesis.

## Abstract (web summary)

MRTF and TAZ are mechanosensitive transcriptional coactivators, but how they functionally interact is not clear. Here the authors show that MRFT and TAZ exhibit multilevel crosstalk in expression, transport and transcriptional activity; furthermore, TAZ confers sensitivity to TGFβ activation of the smooth muscle actin promoter.

## Graphical abstract

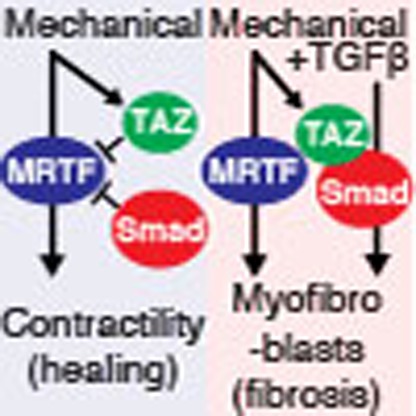

Mechanical inputs (for example, substrate stiffness, stretch, contraction and cell contact disruption) are key regulators of cell fate, phenotype specification, differentiation and growth (reviewed in refs 1, 2, 3, 4, 5). Such mechanical cues are converted into adaptive or maladaptive responses by the cytoskeleton. Accordingly, in addition to its ‘classic' structural roles, the cytoskeleton has emerged as a fate-determining device, which links physical parameters of the environment to gene expression^6^,^7^. The cytoskeleton controls gene transcription primarily through the regulation of the nucleocytoplasmic shuttling of mechanosensitive transcriptional co-activators. The most important representatives of these are the Rho pathway effector myocardin-related transcription factor (MRTF)^6^,^8^ and the Hippo pathway target, WW domain-containing proteins, TAZ and YAP^9^,^10^. Intriguingly, the regulation of the nuclear accumulation of MRTF and TAZ/YAP show striking similarities; for example, nuclear uptake of both is strongly promoted by enhanced matrix stiffness or cellular tension^11^,^12^, disruption of intercellular contacts^13^,^14^,^15^, reduced cell density or peripheral location of cells in a monolayer^16^,^17^. At the molecular level, elevated F-actin content and myosin-based contractility have been associated with nuclear accumulation of both MRTF^13^,^18^ and TAZ/YAP^11^,^19^,^20^,^21^.

The molecular mechanisms through which the cytoskeleton impacts the nuclear traffic of MRTF are well understood. The N-terminal RPEL motif of MRTF can bind G-actin, which masks MRTF's nuclear localization signal. On actin polymerization G-actin dissociates from MRTF, facilitating the nuclear uptake and reducing the nuclear efflux of this protein^22^. Once in the nucleus, MRTF binds serum response factor (SRF), and the complex drives gene expression through the CC(A/T)-richGG cis-element, known as the CArG box^23^,^24^. Since many of the target genes are cytoskeletal proteins, MRTF is a central molecule through which the cytoskeleton regulates the expression of its own components. Previous work by us and others have shown that cytoskeleton remodelling-induced MRTF translocation plays crucial roles in phenotypic shifts including epithelial–mesenchymal/–myofibroblast transition (EMT/EMyT)^13^,^25^,^26^ and fibroblast–myofibroblast transition^27^,^28^,^29^,^30^, key processes in the pathogenesis of organ fibrosis and cancer^31^,^32^. We have shown that both disruption of the intercellular contacts—via the consequent Rho/Rac activation^13^,^33^—and transforming growth factor beta (TGFβ) signalling are necessary for EMyT (two-hit paradigm)^25^, indicating a strong synergy between mechanical and humoral factors in the regulation of plasticity. Indeed, MRTF can bind the TGFβ effector Smad3, and this interaction modulates the transcriptional effects of both proteins^25^,^34^,^35^. Interestingly, TAZ/YAP can also bind Smad3 and act as Smad2/3 nuclear retention factors^36^.

Although TAZ/YAP are major mechanosensitive regulators of organ size, contact inhibition of proliferation and EMT^37^ with recognized roles in the pathogenesis of cancer^38^,^39^ and organ fibrosis^40^,^41^,^42^,^43^, the mechanisms underlying their cytoskeletal control remains enigmatic. The ‘canonical' pathway regulating these factors is the constitutive activity of Hippo kinases, which keep TAZ/YAP phosphorylated, thereby facilitating their cytosolic retention. Disassembly of cell junctions or polarity complexes inhibits the Hippo cascade, leading to TAZ/YAP dephosphorylation and nuclear entry^44^. In the nucleus they associate with various transcription factors, predominantly with members of the TEAD family^45^. While enhanced F-actin polymerization was reported to alter the phosphorylation of Hippo kinases^19^,^46^, the cytoskeletal regulation of TAZ/YAP traffic was shown to be, in part, Hippo-independent^11^,^21^.

The strong similarity in the regulation of the nucleocytoplasmic distribution of MRTF and TAZ/YAP prompted us to investigate whether these key mechanotransducers might interact. In fact we noted that the MRTF C terminus contains a conserved WW-binding motif (PPXY). Therefore, we asked whether MRTF and TAZ might associate (potentially in a WW domain-dependent manner) and whether such interaction might alter the traffic and/or activity of either factor. We also sought to determine if MRTF might confer F-actin sensitivity to TAZ trafficking through a piggyback-type mechanism. We concentrated on TAZ as a potential MRTF partner because our earlier studies implicated TAZ^17^ and MRTF^25^ in EMyT. Our results show that MRTF and TAZ indeed associate partly through a WW-dependent mechanism. While MRTF does not escort TAZ into the nucleus, their interaction has profound functional consequences in terms of their traffic, mobility, expression and transcriptional activity. Moreover, the interactions of TAZ with MRTF or Smad3 constitute a switch, which differentiates between mechanical versus mechanical+chemical effects, thereby integrating context-dependent transcriptional programmes.

## Results

### MRTF and TAZ associate partly via MRTF's WW-binding motif

**Figure 1:**
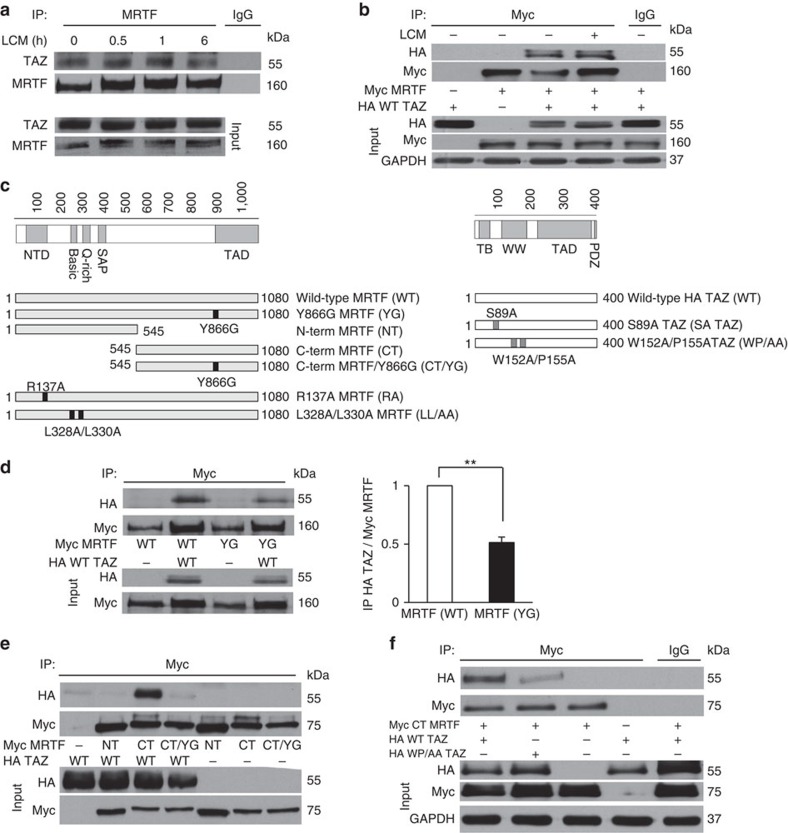
MRTF and TAZ interact partially through the MRTF C terminus and the TAZ WW domain. (**a**) LLC-PK1 cells were incubated under low-calcium conditions (LCM) for the indicated times. Immunoprecipitation (IP) with anti-MRTF reveals that endogenous TAZ specifically co-precipitates with MRTF. (IgG denotes isotope-matched nonspecific antibody.). (**b**) Lysates from cells transfected with Myc-MRTF, HA-TAZ or a combination of both were left untreated or exposed to LCM for 30 min and subjected to IP with anti-Myc. HA-TAZ associates specifically with Myc-MRTF. (**c**) Schematic representation of single or double point mutations in the full-length or truncated proteins used in the study. (**d**) Western blot analysis following co-IP of HA-TAZ with either full-length WT MRTF or YG MRTF. Note that YG MRTF exhibits significantly reduced TAZ binding. Co-precipitating HA-TAZ was normalized to precipitated MRTF and total HA-TAZ expression. Error bars are ±s.e.m.; **P<0.01; Student's *t*-test. (**e**) The N terminus (NT), C terminus (CT) or the C terminus YG mutant (CT/YG) of Myc-MRTF was co-transfected with WT HA-TAZ and their interaction was analysed by co-IP with anti-Myc. Note the strong association of TAZ with the MRTF CT and the loss of association with CT/YG. (**f**) HA-tagged WT or WW mutant TAZ (WP/AA) was co-transfected with MRTF CT, as indicated. Lysates were precipitated with anti-Myc or isotype-matched control antibody (IgG) and probed for the indicated proteins. All immunoblots (**a**,**b**,**d**–**f**) are representative of *n*=3 independent experiments.

To address whether MRTF and TAZ might interact either constitutively or on stimulation, we immunoprecipitated MRTF from resting or low-calcium medium (LCM)-treated cells. The latter uncouples intercellular contacts and activates Rho, stimuli that induce nuclear translocation of both MRTF^13^ and TAZ^14^. A substantial amount of TAZ was present in the MRTF immunoprecipitate of resting cells, which did not significantly change on LCM stimulation (Fig. 1a). To verify complex formation and allow structural characterization of this interaction, cells were transfected with HA-TAZ and Myc-MRTF. Anti-Myc antibody efficiently and specifically pulled down HA-TAZ from Myc-MRTF-transfected cells in the absence or presence of LCM (Fig. 1b). To test if the putative WW-binding motif (PPRY) in the C-terminal half of MRTF could contribute to the observed association, we generated various constructs encoding the full-length MRTF with a point mutation (Y866G, designated as YG) and truncation mutants encoding the N-terminal and C-terminal half with or without the YG mutation (Fig. 1c). Normalized to the precipitated MRTF, there was >50% reduction in the association of HA-TAZ with the full-length YG mutant compared with wild-type (WT) MRTF (Fig. 1d). Nonetheless, the association was not fully lost indicating that the WW-binding motif is a significant but not exclusive contributor to the MRTF/TAZ interaction. The N-terminal half failed to interact with HA-TAZ, suggesting that, at least in isolation, this portion of MRTF (containing the actin-binding RPEL motifs and the SRF-binding site) is insufficient for stable interaction with TAZ (Fig. 1e). In contrast, the C-terminal half exhibited strong association with HA-TAZ, which was drastically reduced in the YG mutant (Fig. 1e). Point mutations in the WW motif (W152A/P155A, designated WP/AA) of TAZ (Fig. 1c), which are expected to weaken the binding capacity of this domain^47^, markedly reduced the association between TAZ and the MRTF C terminus (Fig. 1f). Taken together, MRTF and TAZ can form a complex and the C-terminal WW via binding motif of MRTF plays a key role in this interaction.

### MRTF does not confer actin sensitivity to TAZ

**Figure 2:**
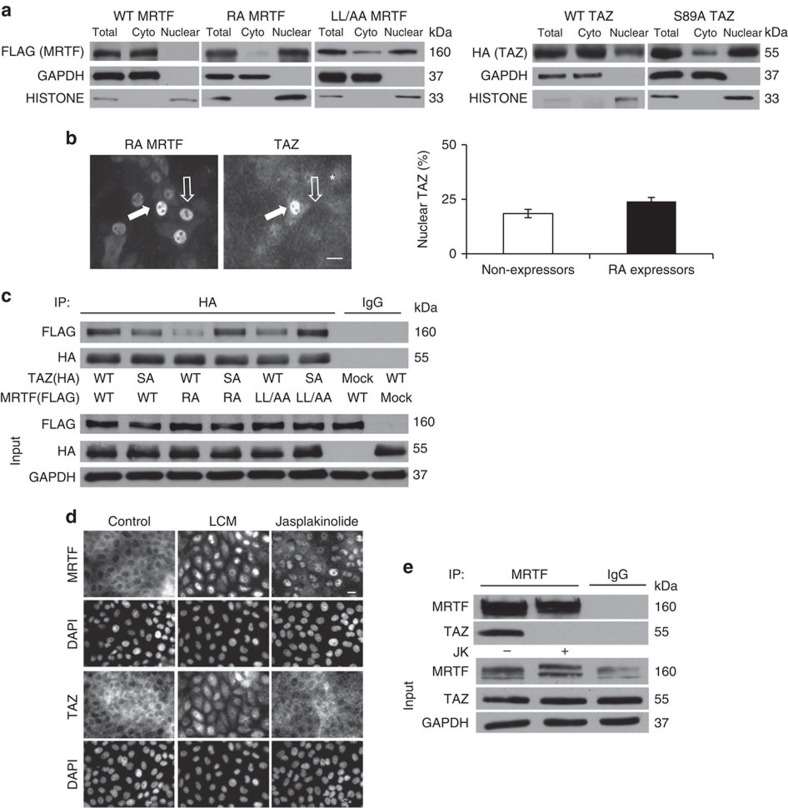
Nuclear translocation of MRTF and TAZ are not directly coupled. (**a**) LLC-PK1 cells were transfected with FLAG-tagged MRTF or HA-tagged TAZ constructs. The distribution of the tagged proteins in the nuclear and cytosolic compartments was analysed by cell fractionation followed by western blotting. Representative blots are shown for *n*=4 experiments. (**b**) Cells plated on glass coverslips were transfected with FLAG-tagged RA MRTF and stained with anti-FLAG and anti-TAZ. Scale bar, 10 μm. The percentage of cells with nuclear TAZ was quantified in nuclear RA expressors (black column) and in untransfected cells (open column) as endogenous control on the same coverslips (at least 100 cells per condition in *n*=4 experiments; error bars, ±s.e.m.) (RA MRTF was nuclear in 93% of the cells, see Supplementary Fig. 1). The white arrow indicates a cell counted as positive for both nuclear MRTF and TAZ, whereas the black arrow denotes a cell counted as positive for nuclear MRTF but negative for nuclear TAZ. (**c**) Association profile of WT and mutant MRTF and TAZ constructs. Cells were transfected with FLAG-tagged WT (cytosolic) or RA (fully nuclear) or LL/AA (predominantly nuclear) MRTF constructs along with WT or active (SA, largely nuclear) HA-TAZ. After immunoprecipitation (IP) with anti-HA the precipitates and total lysates were probed for the indicated proteins (*n*=3). (**d**) Immunofluorescence analysis of MRTF and TAZ after confluent cells were treated with LCM or 0.5 μM JK for 30 min (*n*=5; scale bar, 10 μm). (**e**) JK disrupts the association of MRTF and TAZ. Cells were exposed to vehicle or 0.5 μM JK for 30 min and lysed. The resulting lysates were immunoprecipitated with anti-MRTF antibody, and the precipitates were probed for MRTF and TAZ (*n*=3).

Having established that MRTF and TAZ can associate, we asked if MRTF might confer actin sensitivity to TAZ transport via a piggyback mechanism. To address this, we generated a FLAG epitope-tagged MRTF mutant (R137A, RA), which is deficient in G-actin binding and thus localizes preferentially to the nucleus^4^, and assessed its impact on TAZ distribution. Cell fractionation (Fig. 2a) and immunostaining (Supplementary Fig. 1a) confirmed that WT MRTF was almost entirely cytosolic, whereas RA MRTF was fully nuclear. While nuclei with very high RA MRTF levels occasionally showed TAZ accumulation, the distribution of endogenous TAZ did not match nuclear MRTF accumulation (Fig. 2b). Overall, ≈25% of RA nuclear expressors exhibited TAZ accumulation in the nucleus, which was only marginally higher than that of neighbouring cells without RA expression (≈20%). These findings did not lend support for the piggyback hypothesis; nonetheless it was conceivable that the incapability of RA MRTF to chaperone TAZ into the nucleus could be due to its inability to bind TAZ. To address this, we co-expressed FLAG-tagged WT and RA MRTF with HA-tagged WT TAZ or S89A TAZ (labelled as SA TAZ, an active, dephospho-mimic mutant), and performed immunoprecipitations with anti-HA antibody (Fig. 2c). As expected from the reciprocal immunoprecipitations (Fig. 1b,d), WT TAZ readily associated with WT MRTF, while SA TAZ exhibited reduced binding. Similarly, the RA mutation drastically decreased the association between WT TAZ and MRTF (Fig. 2c). These findings could be consistent with a reduced affinity of RA MRTF or SA TAZ for the corresponding WT partners, but a more plausible explanation is that the constructs show reduced association if they are localized to different compartments. Indeed, SA TAZ, which also localizes predominantly to the nucleus (Fig. 2a, right panel), exhibited strong binding to RA MRTF (Fig. 2c). To substantiate this conclusion, we used another MRTF mutant (L328A/L330A, designated LL/AA) which is deficient in nuclear efflux and therefore also shows preferential (albeit not exclusive) nuclear accumulation^48^ (Fig. 2a). LL/AA exhibited a similar pattern of interaction with TAZ as the RA mutant, except it bound slightly more WT TAZ, in accordance with its higher cytosolic level (Fig. 2c). Thus, two structurally different mutations both mitigated the association with WT, but not with SA TAZ. In short, these molecules interacted efficiently when both were in the same compartment but not when directed to different ones, reinforcing that their translocation is not coupled.

To examine the movement of endogenous MRTF and TAZ in response to various stimuli, cells were exposed either to LCM or jasplakinolide (JK), a potent actin-polymerizing agent, and their localization visualized by immunostaining (Fig. 2d). LCM (30 min) caused marked nuclear translocation of both MRTF and TAZ. In contrast JK triggered massive nuclear accumulation of MRTF, but failed to affect TAZ distribution at 30 min. Indeed, corresponding co-immunoprecipitations revealed that JK uncoupled MRTF from TAZ (Fig. 2e). These findings not only indicate that MRTF and TAZ translocate independently to the nucleus but also suggest that actin polymerization *per se* is not sufficient for TAZ translocation (see Discussion).

### TAZ mitigates the nuclear accumulation of MRTF

**Figure 3:**
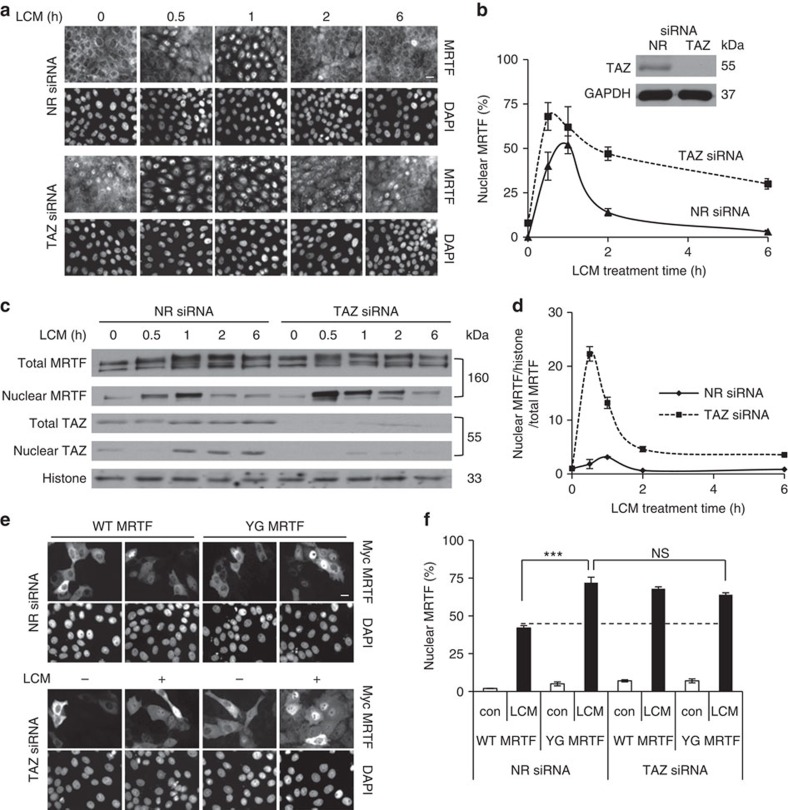
MRTF downregulation potentiates stimulus-induced nuclear accumulation of TAZ in a WW-binding domain-dependent manner. (**a**,**b**) Cells transfected with NR or TAZ siRNA for 48 h were exposed to LCM treatment for the indicated times, stained for MRTF and 4,6-diamidino-2-phenylindole (DAPI), and the percentage of cells with nuclear MRTF was determined (200 cells per condition; *n*=3; scale bar, 10 μm). (Inset) Verification of TAZ silencing by western blot. (**c**,**d**) Nuclear extracts were prepared from cells transfected and treated as in **a** and probed with anti-MRTF and anti-TAZ. Equal loading was verified using anti-histone antibody. Nuclear MRTF was quantified by densitometry, normalized to histones and total MRTF levels, and expressed as fold change compared with the untreated control (*n*=3). (**e**) Cells were co-transfected with Myc-MRTF (WT or YG) and either NR or TAZ siRNA. Following LCM treatment (30 min) the localization of the MRTF constructs was examined using anti-MRTF antibody (*n*=4). Scale bar, 10 μm (**f**) Percentage of cells with nuclear MRTF under conditions shown in **e**. For **b**,**d** and **f** error bars denote ±s.e.m.; ***P<0.001, NS, not significant.

While TAZ and MRTF do not co-translocate to the nucleus, their association raised the possibility that they might impact each other's transport. To test this we first followed LCM-induced nuclear accumulation of MRTF in cells transfected with a non-related control (NR) siRNA or TAZ siRNA by immunostaining (Fig. 3a,b and Supplementary Fig. 1b) and western blotting of nuclear extracts (Fig. 3c,d). In cells transfected with NR siRNA, the LCM-triggered nuclear accumulation of MRTF peaked between 30 and 60 min, after which MRTF returned to the cytosol in an hour (Fig. 3b,d), as in our previous studies^25^. Elimination of TAZ did not change MRTF localization in resting cells, (cytosolic in >90% of the cells) but had a major impact after stimulation. LCM induced more rapid and longer-lasting nuclear MRTF accumulation in a higher percentage of TAZ siRNA-transfected cells than in NR siRNA-treated controls (Fig. 3a,b). Measuring total nuclear MRTF protein as a function of time (Fig. 3c,d) indicated that TAZ silencing resulted in a 10-fold rise in nuclear MRTF compared with the NR siRNA-treated controls after 30-min LCM stimulation. The peak was not only larger but occurred earlier. Subsequently, nuclear MRTF content decreased in TAZ-depleted cells as well, but remained higher than the control over the entire time course. Of note, in control cells LCM-induced translocation of MRTF preceded that of TAZ, confirming their separate nuclear entry. To assess whether a WW motif-dependent interaction (Fig. 1) contributes to the effect of TAZ on MRTF transport, we expressed Myc-tagged WT or YG MRTF and quantified LCM-induced MRTF translocation in control and TAZ-downregulated cells (Fig. 3e,f). After 30-min LCM stimulation, the percentage of cells with strong nuclear Myc staining was twice as high (≈80%) in YG as in WT expressors. Moreover, TAZ silencing doubled the LCM-triggered nuclear accumulation of the WT construct, but failed to affect that of YG, that is, it abolished the difference in translocation between these constructs. These results indicate that TAZ mitigates LCM-induced nuclear accumulation of MRTF in a WW motif-dependent manner.

### MRTF maintains TAZ expression but hinders TAZ translocation

**Figure 4:**
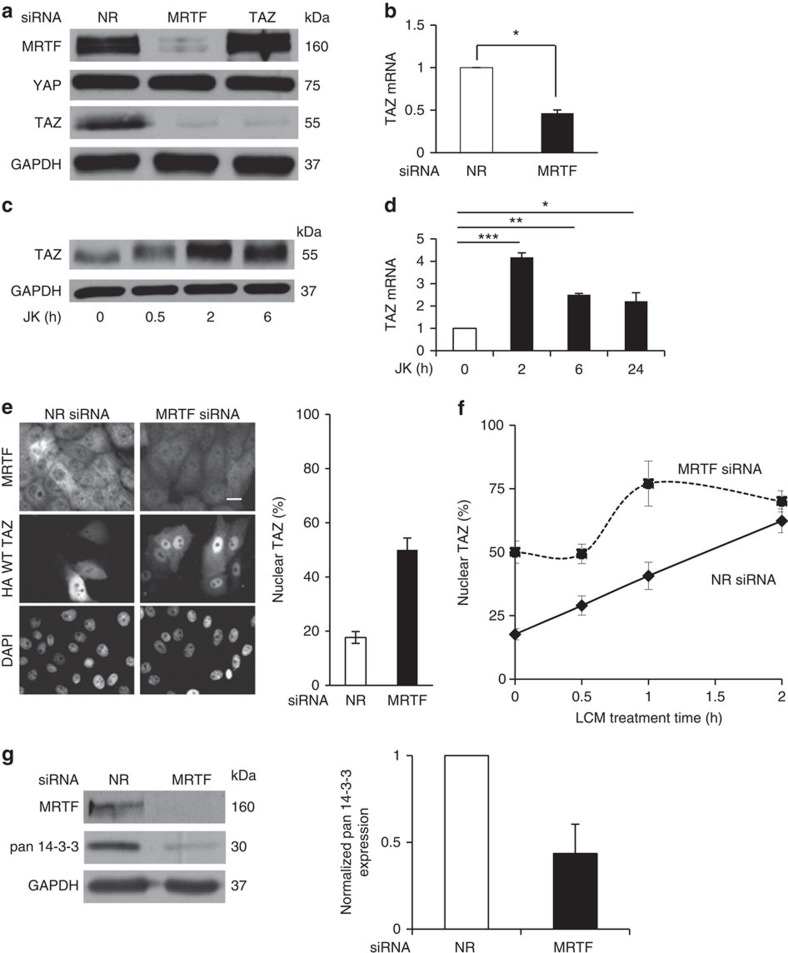
MRTF regulates TAZ expression and localization. (**a**) Cells transfected with NR or specific siRNAs for 48 h were analysed by immunoblotting for expression of the indicated proteins. (**b**) The effect of MRTF silencing on TAZ mRNA levels (*n*=3). (**c**) Enhanced actin polymerization increases TAZ expression. Confluent monolayers were treated with 0.5 μM JK for the indicated times and TAZ protein expression was determined by western blotting. (**d**) Cells were treated with JK as indicated and analysed for TAZ mRNA. (**e**) Cells co-transfected for 48 h with HA-TAZ and NR or MRTF siRNA were doubly stained for HA and MRTF (left panel). Percentage of cells with nuclear HA-TAZ was quantified (right panel). Scale bar, 10 μm. (**f**) Immunofluorescence analysis of cells transfected with NR or MRTF siRNA, followed by treatment with LCM for the indicated times. The percentage of cells showing nuclear TAZ accumulation was plotted as a function of the treatment time. (**g**) MRTF was silenced as in **e** and the expression of 14-3-3 proteins was determined by western blotting and quantified by densitometry. Western blots (**a**,**c**,**g**) were repeated a minimum of three times. Error bars in **b**,**d**,**f**,**e** and **g** represent ±s.e.m.; *P<0.05; **P<0.01; ***P<0.001 by Student's *t*-test or one-way analysis of variance.

We wished to test if the inhibition is mutual, that is, if MRTF can hinder TAZ transport. To assess this we downregulated MRTF and unexpectedly found that MRTF silencing caused a robust decrease (>80%) in TAZ expression without affecting YAP (Fig. 4a). In contrast, TAZ silencing had no effect on MRTF expression (Fig. 4a). MRTF knockdown resulted in a 50% reduction in TAZ mRNA (Fig. 4b), a finding consistent with the presence of a CArG box in the TAZ promoter (see Discussion). We argued that if MRTF can drive TAZ expression then increased actin polymerization, a key trigger for MRTF translocation to the nucleus, should stimulate TAZ expression. Indeed, JK markedly enhanced TAZ protein expression (Fig. 4c) and mRNA levels (Fig. 4d) within 2 h. These findings imply that MRTF is a critical regulator of TAZ expression.

To overcome the transcriptional effect of MRTF on endogenous TAZ, we expressed HA-TAZ (driven by the cytomegalovirus (CMV) promoter) in cells co-transfected with NR or MRTF-specific siRNA. MRTF downregulation caused a 2.5-fold increase in the nuclear localization of HA-TAZ (Fig. 4e). Moreover, LCM treatment caused faster HA-TAZ translocation in cells transfected with MRTF siRNA than with NR siRNA (Fig. 4f). These findings suggest that MRTF promotes the cytosolic retention of TAZ. Since 14-3-3 proteins were implicated as major TAZ retention factors^49^ we tested the impact of MRTF on these. Downregulation of MRTF strongly reduced 14-3-3 expression (Fig. 4g), suggesting that MRTF might support TAZ retention at least partly via 14-3-3.

**Figure 5:**
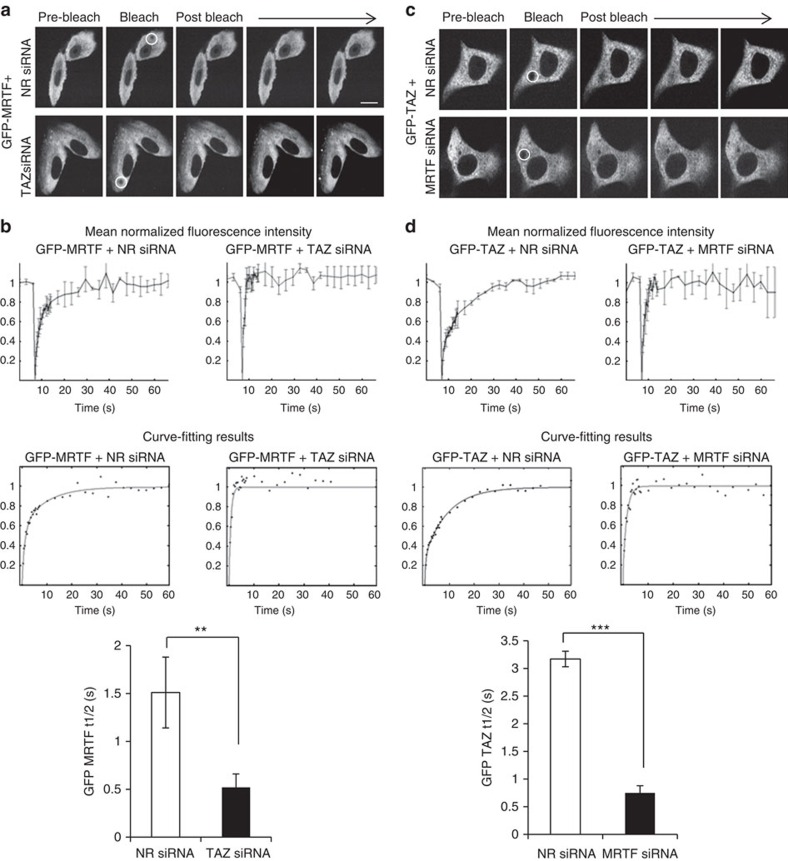
TAZ and MRTF mutually inhibit each other's cytosolic mobility. (**a**) Cells were co-transfected with GFP-MRTF and NR or TAZ siRNA, and FRAP experiments were performed on the cytosolic GFP-MRTF pool (representative images). Bleached regions of interest are circled. (**b**) Recovery curves (+/−s.d.) and the corresponding curve fitting. Data were normalized to the pre-bleach fluorescence levels. The bar diagram shows the halftime of recovery (*n*=15 per condition). (**c**) Cells were co-transfected with GFP-TAZ and NR or MRTF siRNA, and FRAP experiments were performed on the cytosolic GFP-TAZ pool (representative images). (**d**) Analysis was performed as in **b** (*n*=15 per condition). Error bars represent ±s.e.m.; **P<0.01; ***P<0.001; scale bar, 10 μm.

We then asked if TAZ and MRTF impede each other's cytosolic mobility and thereby traffic. We first transfected cells with green fluorescent protein (GFP)-MRTF along with NR or TAZ-specific siRNA, and performed fluorescence recovery after photobleaching (FRAP) experiments (Fig. 5a,b). The halftime for recovery of GFP-MRTF was twice as long in control as in TAZ-depleted cells, indicating that TAZ significantly reduces MRTF's mobility (Fig. 5b). In the inverse experiment cells were transfected with NR or MRTF-specific siRNA along with GFP-TAZ and the mobility of the latter was measured (Fig. 5c,d). MRTF downregulation caused a fourfold increase in the mobility of TAZ (Fig. 5d). Taken together, these experiments show that MRTF is needed for TAZ expression and inhibits the basal and stimulus-induced nuclear localization/accumulation and the cytosolic mobility of TAZ. Conversely, TAZ does not affect MRTF expression or basal distribution but suppresses its mobility and the stimulus-induced nuclear accumulation. Thus, the two proteins reciprocally mitigate each other's nuclear translocation.

### Transcriptional interactions between MRTF and TAZ

**Figure 6:**
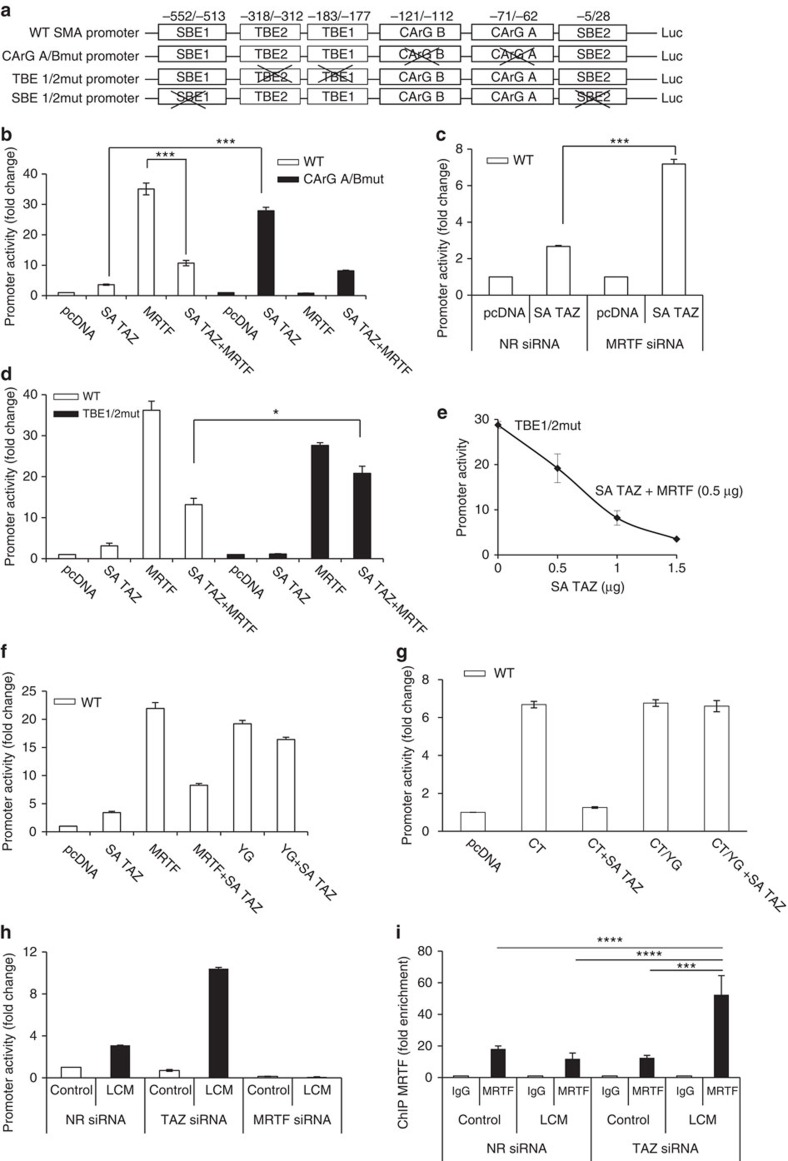
Transcriptional interactions between MRTF and TAZ on WT and mutant SMA promoters. (**a**) WT and mutant SMA promoter luciferase constructs used in this study. (**b**) Cells were co-transfected with WT or CArGmut promoter firefly luciferase construct and renilla-TK reporter along with either empty vector control (pcDNA) or the indicated expression plasmids. In all subsequent experiments luciferase activities were determined 24 h post transfection and firefly/renilla ratios were normalized to the pcDNA control. (**c**) NR or MRTF siRNA were transfected along with WT SMA promoter and pcDNA or SA HA-TAZ expression plasmid. (**d**) The WT or TBEmut SMA promoter was co-transfected with the indicated plasmids. (**e**) Titration of the effect of MRTF (0.5 μg) on TBEmut promoter using increasing doses (0–1.5 μg) of TAZ. (**f**,**g**) WW-binding domain interactions are important for the TAZ-induced inhibition of MRTF-mediated SMA promoter activation. The WT SMA promoter was transfected with full-length WT or YG MRTF (**f**) or CT and CT/YG MRTF (**g**) without or with SA TAZ. (**h**) Cells were co-transfected with the WT SMA promoter along with NR, TAZ or MRTF siRNA, followed by exposure to control or LCM media. (**i**) Cells were transfected with NR or TAZ siRNA (siTAZ #2) then incubated in control or LCM media for 24 h. Subsequently, ChIP was performed using anti-MRTF antibody and the abundance of the endogenous SMA promoter in the precipitated DNA was determined by qPCR (*n*=3). All luciferase assays (**b**–**h**) were performed a minimum of three times. Error bars (**b**–**i**) are ±s.e.m.; *P<0.05, ***P<0.001 and ****P<0.0001 derived by one-way analysis of variance.

Since several conditions translocate both MRTF and TAZ to the nucleus, it is important to define how the two co-activators affect each other's transcriptional effect. To address this we overexpressed TAZ and MRTF alone or in combination and assessed their effect on WT and mutant promoter constructs (Fig. 6a). Initially we studied this question in the context of the SMA promoter, since SMA expression is a hallmark of myofibroblast transition, and the SMA promoter contains adjacent CArG boxes and TEAD-binding elements (TBE, also called MCAT), as well as Smad-binding elements (SBE). In agreement with our previous data^17^,^25^, overexpression of active (nuclear) SA TAZ caused a significant but modest (3- to 4-fold) stimulation, while overexpression of MRTF robustly activated (25- to 30-fold) the WT SMA promoter (Fig. 6b). These effects were mediated via the corresponding cis-elements since mutation of TBEs (TBEmut) or the CArG boxes (CArGmut) eliminated the respective stimulatory effect of TAZ and MRTF (Fig. 6b,d). Interestingly, when expressed together, SA TAZ strongly inhibited the stimulatory effect of MRTF on the WT promoter (Fig. 6b). Conversely, SA TAZ stimulated the CArGmut promoter 7-fold more potently than the WT promoter (Fig. 6b), and overexpression of MRTF potently inhibited this strong stimulatory effect (Fig. 6b). Together these findings indicate that both MRTF and TAZ can stimulate the SMA promoter through different cis-elements, but they mutually inhibit each other's transcriptional effect. Furthermore, the fact that TAZ becomes a stronger activator on the CArGmut, (that is, in the absence of basal MRTF/SRF recruitment to the promoter) suggests that MRTF can inhibit the transcriptional effect of TAZ most efficiently at the promoter itself. Of note, this inhibitory action is distinct from any additional effect of MRTF on TAZ transport. In keeping with these results, MRTF knockdown potentiated the effect of SA TAZ overexpression on the WT SMA promoter (Fig. 6c). Finally, analysis of the combinatorial effect on the TBEmut promoter revealed that SA TAZ overexpression had a weak inhibitory effect on the MRTF-provoked response (Fig. 6d); nonetheless higher SA TAZ doses counteracted the effect of MRTF even on this promoter (Fig. 6e). These findings indicate that TAZ-mediated inhibition of MRTF's effect is facilitated by the presence of TBE, although TBE is not an absolute requirement.

Next we tested whether the inhibitory action of TAZ on MRTF requires the WW-binding motif of MRTF (Fig. 6f–g). YG full-length MRTF was nearly as efficient in driving the SMA promoter as the WT but was essentially insensitive to the inhibitory action of TAZ (Fig. 6f). Similar results were obtained for the C-terminal MRTF fragment (Fig. 6g). Conversely, WT MRTF exerted significantly stronger inhibitory effect on the TAZ-induced activation of the CArGmut promoter than YG MRTF (Supplementary Fig. 2). Thus, the WW-binding motif-dependent interaction between MRTF and TAZ is central for the effect of these proteins on each other's transcriptional activity. We then investigated the transcriptional effects in the context of cell stimulation (as opposed to factor overexpression). Downregulation of TAZ strongly potentiated the LCM-induced, modest activation of the SMA promoter, an effect that required MRTF (Fig. 6h). Finally, we tested if TAZ impacts MRTF binding in the context of the endogenous SMA promoter. Chromatin immunoprecipitation (ChIP) revealed that on LCM stimulation the endogenous SMA promoter bound significantly more MRTF in TAZ-depleted than controls cells (Fig. 6i).These results confirm that under these stimulatory conditions TAZ mitigated the access of MRTF to the natural SMA promoter.

### TAZ confers Smad3 sensitivity to the SMA promoter via SBEs

**Figure 7:**
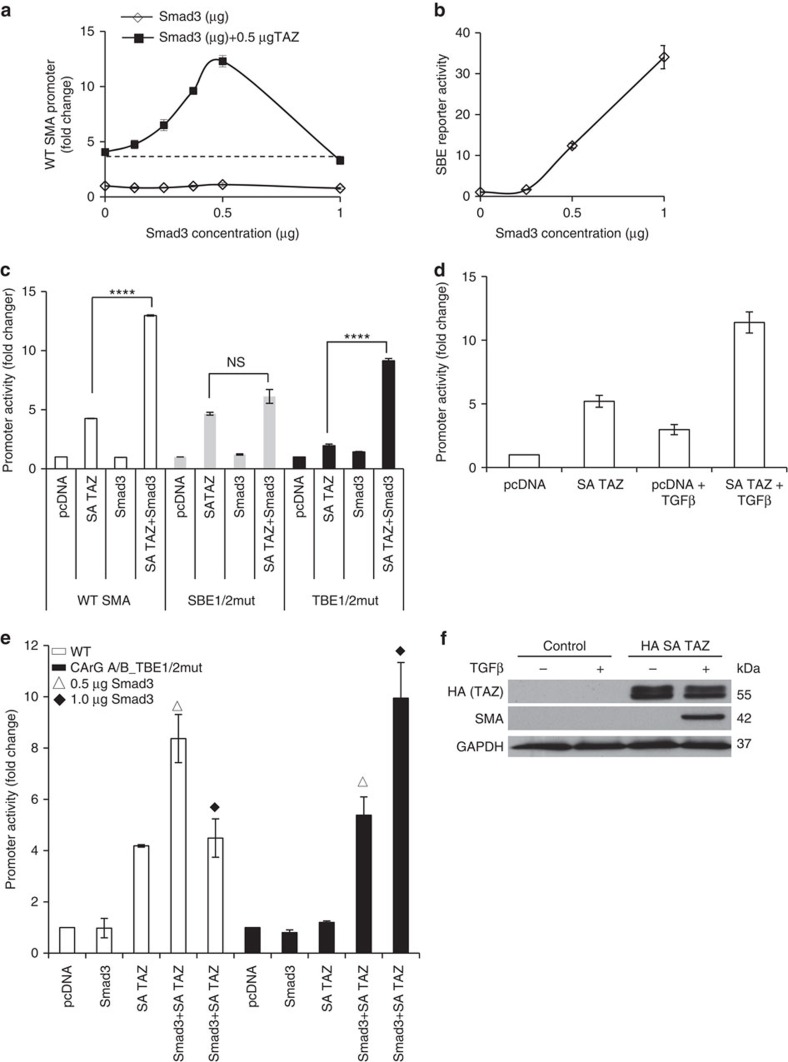
TAZ confers Smad3 sensitivity to the SMA promoter. (**a**) Cells were transfected with the WT SMA promoter and increasing concentrations of Smad3 alone or along with a constant amount (0.5 μg) of SA TAZ (*n*=3). (**b**) A varying dose of Smad3 was co-transfected with the SBE4-luc reporter construct (*n*=3). (**c**) SBE but not TBE is critical for the Smad3 sensitivity of the SMA promoter. Luciferase activity was measured in cells co-transfected with the indicated promoter constructs and expression plasmids. Data were normalized to the activity of WT SMA promoter in empty vector-transfected cells (*n*=5). (**d**) Cells were transfected with the WT SMA promoter and either empty plasmid (pcDNA) or SA TAZ. Following treatment with or without TGFβ, luciferase activity was measured (*n*=3). (**e**) The WT or CArG A/B_TBE1/2mut SMA promoter construct was transfected with Smad3 (0.5 or 1 μg, as indicated), SA TAZ (0.5 μg) or a combination of these, and luciferase activity was determined (*n*=3). (**f**) SA TAZ overexpression enables TGFβ to induce SMA expression. Cells were transfected with control vector or HA-tagged SA TAZ for 24 h and then left untreated or exposed to TGFβ, as indicated, for an additional 48 h. Western blotting was then performed for the indicated proteins (*n*=3). (**a**–**e**) Error bars represent ±s.e.m.; ****P<0.0001; NS, not significant using one-way analysis of variance.

Smad3 is a key mediator of TGFβ signalling, and the SMA promoter contains two SBEs. However, the role of Smad3 in SMA expression is controversial. We have previously shown that Smad3 alone does not activate the SMA promoter, and it strongly inhibits the stimulatory effect of MRTF^25^. Given that Smad3 and TAZ can interact, and TAZ can act as a Smad3 nuclear retention factor^36^, we asked if TAZ could confer Smad3 sensitivity to the SMA promoter. In agreement with our previous studies^25^, overexpression of Smad3 alone failed to drive the SMA promoter (Fig. 7a) in a concentration range in which it strongly (15- to 30-fold) stimulated an SBE reporter (Fig. 7b). To assess the impact of active TAZ, we co-transfected cells with a constant amount of SA TAZ along with varying amounts of Smad3, and measured the activity of the SMA promoter. The presence of TAZ enabled Smad3 to drive the SMA promoter in a concentration-dependent and biphasic manner. At the most effective concentration (0.5 μg DNA in our system) Smad3 induced a >3-fold activation of the promoter over and above the stimulatory effect of TAZ itself (Fig. 7a). At higher concentrations this synergy was lost (see below). To discern if the effect of Smad3 was mediated through TBE or SBE, we expressed Smad3 and SA TAZ alone or in combination, and tested their effects on the WT, SBE-mutated (SBEmut) and TBE-mutated promoters (Fig. 7c). The potentiating effect of Smad3 seen with the TBEmut was lost on the SBEmut, indicating that TAZ confers Smad3 sensitivity to the SMA promoter through SBE. Moreover, SA TAZ enabled TGFβ to significantly potentiate the SMA promoter, that is, a natural Smad3 activator had the same effect as Smad3 overexpression (Fig. 7d). We next addressed why the stimulatory effect of Smad3 was lost at higher doses. We surmised that the inhibitory effect of high Smad3 doses on endogenous MRTF counteracted the stimulatory effect through SBE. To test this, we generated a double-mutant reporter, in which both the CArG and TBE elements were inactivated. Consistent with our hypothesis, the activity of this construct continued to increase at a higher Smad3 dose (Fig. 7e). Next, we argued that if Smad3/TAZ represents a potent input to activate SMA expression, then overexpression of active TAZ should enable TGFβ to induce SMA protein expression. Indeed, while neither TGFβ nor SA TAZ alone was able to elicit SMA expression, their combination did so (Fig. 7f).

### Context-dependent interplay between MRTF, TAZ and Smad3

Our experiments presented so far suggest an intriguing scenario. Namely, in the context of the SMA gene TAZ may act as an inhibitor (by interfering with MRTF) but may also work as an activator (partnering with Smad3), depending on the type of stimulation. Thus, TAZ may be a functional switch during contact-dependent or mechanical versus mechanical+TGFβ-induced signalling. Accordingly, the aim of the following studies was twofold: to test this switch hypothesis under well-defined, single- or double-hit conditions, and to generate insight into the underlying mechanisms by exploring the interplay among the three factors.

**Figure 8:**
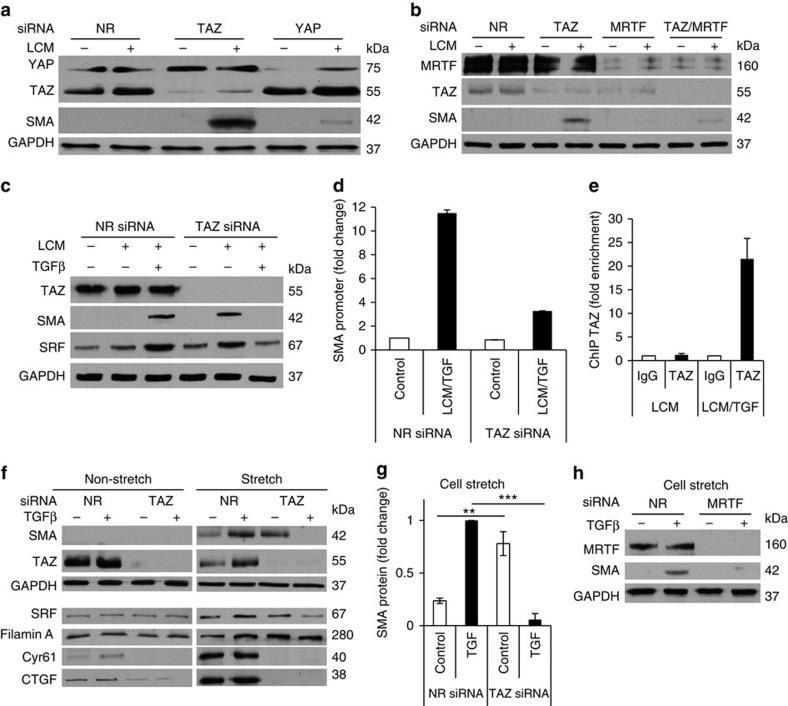
Context-dependent switch in mechanical versus mechano/chemical signalling assigns opposite role for TAZ under mechanical (single) versus mechanical+chemical (double) stimulation. (**a**–**e**) Single or double stimulation in the context of LCM-induced cell contact disassembly −/+TGFβ treatment (**a**) TAZ downregulation enables LCM to induce SMA expression. Cells were transfected with NR, TAZ-specific (#1) or YAP-specific siRNA for 24 h and then exposed to control or LCM medium for an additional 48 h. Cell lysates were probed for SMA and the other indicated proteins. (**b**) MRTF is required for the expression of SMA on TAZ downregulation and LCM treatment. Cells were transfected with NR or specific siRNAs against TAZ (#2), MRTF or their combination, and treated and analysed for the indicated proteins as in **a**. (**c**) TAZ downregulation (siTAZ #2) inhibits two-hit (LCM+TGFβ)-induced SMA protein expression. Cells were transfected with NR or TAZ siRNA followed by 48 h treatment with LCM, TGFβ or the combination. Cell lysates were subjected to western blot analysis for the indicated proteins. (**d**) Cells were co-transfected with the WT SMA promoter and either NR or TAZ siRNA (siTAZ #2). Luciferase activity was measured following treatment with control or LCM+TGFβ (*n*=3). (**e**) Confluent monolayers were treated with LCM or LCM+TGFβ for 24 h. ChIP analysis was performed using anti-TAZ or the relevant IgG control and primers for the SMA promoter (*n*=3). (**f**–**h**) Single or double stimulation in the context of stretch −/+TGFβ treatment. TAZ differentially regulates SMA expression on stimulation by stretch or by stretch and TGFβ. Cells grown on Flexcell membranes were transfected with NR or TAZ siRNA (siTAZ #1), treated without or with TGFβ and then further exposed to non-stretch or cyclic stretch conditions (10% stretch at 1 Hz for 6 h). (**f**) Cell lysates were prepared and analysed by western blotting for the indicated proteins. (**g**) The graph depicts the quantitation of SMA expression under stretch conditions. Data are expressed as fold change compared with the level of stretch+TGFβ-induced SMA expression. (**h**) Stretch+TGFβ induction of SMA protein expression is dependent on MRTF. Cells were transfected with NR or MRTF-specific siRNA and were subjected to 6 h of cell stretch in the presence or absence of TGFβ. Lysates were collected and analysed by western blot for the indicated proteins. Western blots in **a**–**c**,**f** and **h** are representative of a minimum of three independent experiments. Error bars in **d**,**e** and **g** denote ±s.e.m.; **P<0.01; ***P<0.001 by pairwise *t*-tests.

First, we used LCM as stimulus, which activates MRTF and TAZ but not Smad3. As we reported earlier, LCM alone did not induce SMA expression^25^,^50^. However, TAZ downregulation enabled LCM to provoke SMA expression (Fig. 8a), in agreement with the inhibitory action of TAZ on MRTF. Of note the impact of TAZ was much stronger than that of YAP (Fig. 8a,b). Importantly, the LCM-induced SMA expression remained fully MRTF-dependent, as verified by MRTF silencing (Fig. 8b). The role of TAZ was very different when LCM was added together with TGFβ. According to our well-established two-hit model neither LCM nor TGFβ alone induced SMA expression while together they elicited a strong response^25^,^50^. The two-hit-provoked SMA expression was abolished by TAZ downregulation (Fig. 8c). Accordingly, TAZ silencing reduced the TGFβ+LCM-induced activation of the SMA promoter (Fig. 8d) in contrast to the stimulatory effect seen when LCM was the sole stimulus (Fig. 6h). Moreover, chromatin immunoprecipitation revealed a large increase in TAZ binding to the endogenous SMA promoter on LCM+TGFβ stimulation, compared with LCM alone (Fig. 8e).

We next tested if a reversal in TAZ function is also present in a genuine mechanotransduction context. Cells were challenged with cyclic stretch, which induced the nuclear translocation of both factors (Supplementary Fig. 3a,b). Stretch was applied alone or in combination with TGFβ. These stimuli also constituted a typical double-hit scheme wherein stretch or TGFβ alone caused no or only marginal SMA expression, but their combination led to a strong response (Fig. 8f,g). The response remained fully MRTF-dependent (Fig. 8h). Again, downregulation of TAZ has a diametrically opposite impact under the two conditions; it stimulates SMA expression on stretch but abolishes it when stretch+TGFβ is applied (Fig. 8f,g). Importantly, this pattern was not restricted to SMA alone. We tested some representative proteins whose gene promoters harbour cis-elements for MRTF, TAZ and Smad3 between −2,000 and +1,000 b from the transcription start site^24^,^51^ (Fig. 8f, lower panels). Remarkably, SRF showed a similar pattern as SMA. Considering that SRF is a master regulator of a large set of cytoskeletal and early genes, this regulatory mode may have widespread influence. Filamin was also induced by TAZ dowregulation although this was observed even in the presence of TGFβ. Taken together, TAZ acts as a mechanochemical switch for a variety of targets. Finally, the expression of CTGF and Cyr61, which are classic TAZ targets, required TAZ under all conditions.

### TGFβ induces redistribution of TAZ between MRTF and Smad3

**Figure 9:**
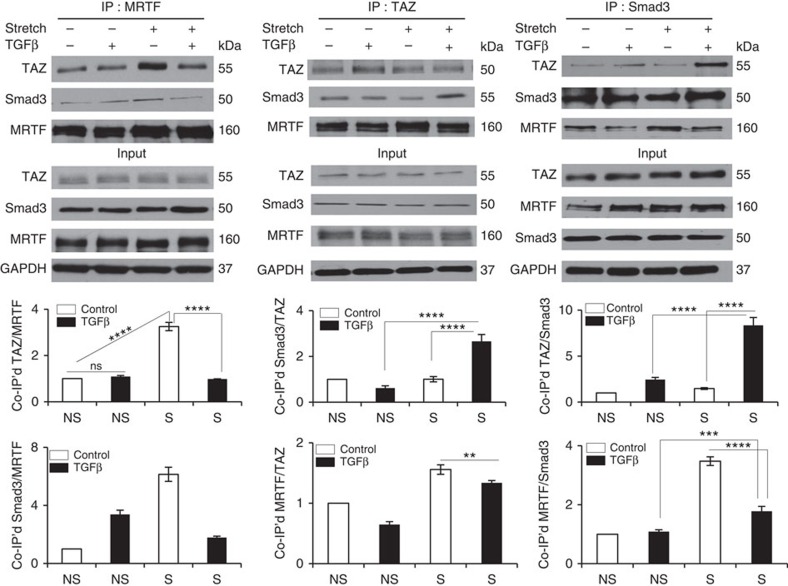
Differential association between MRTF, TAZ and Smad3 on mechanical or/and TGFβ-induced stimulation. Cells were exposed to vehicle or TGFβ, followed by control (non-stretch, NS) or cyclic stretch (S) conditions for 6 h in the presence or absence of TGFβ, as in Fig. 8f. Immunoprecipitations (IPs) were performed on the resulting cell lysates using antibodies against MRTF, TAZ or Smad3, as indicated. The immunoprecipitates and input samples were then probed for the indicated proteins. The graphs show quantification of the corresponding protein–protein associations, normalized to the directly immunoprecipitated protein and using the co-precipitating protein/precipitated protein ratio obtained under non-stretch, unstimulated conditions as unity (*n*=3). Error bars are ±s.e.m.; **P<0.01; ***P<0.001; ****P<0.0001; ns, not significant using one-way analysis of variance.

To explain these observations, we surmised that in the absence of TGFβ, MRTF is in complex with TAZ (and/or Smad3), which inhibits MRTF, while in the presence of TGFβ, TAZ and Smad3 may associate, and one or both may dissociate from MRTF. To test if such redistribution occurs, we conducted co-immunoprecipitation experiments using antibodies against each of the three factors under conditions of non-stretch or stretch −/+TGFβ (Fig. 9). Applied individually, stretch promoted the association of MRTF with TAZ and Smad3, while TGFβ had no or modest effect. Remarkably, TGFβ prevented or reduced the association of MRTF with TAZ or Smad3 in the stretched cells, while significantly increasing the association of TAZ and Smad3. Thus TGFβ counteracted the stretch-induced association of MRTF with TAZ and Smad3, and promoted Smad3/TAZ association instead.

Finally, while MRTF and TAZ acted antagonistically in the context of the SMA promoter where the corresponding cis-elements are adjacent, MRTF potentiated TAZ-mediated responses on TBEs, which are not located in the vicinity of a CArG box. The corresponding experiments are shown and described in the Supplementary Fig. 4.

## Discussion

Our studies indicate structural and functional interaction between two major cytoskeleton-regulated transcriptional co-activators, MRTF and TAZ. We show that these factors associate in a manner partly dependent on the WW-binding motif of MRTF and the WW domain of TAZ. Such PPXY-mediated interaction is functionally significant, because mutation of this motif alters the effect of the two proteins on each other's nucleocytoplasmic traffic and transcriptional activities. A previous study reported that YAP1 and myocardin can co-precipitate in smooth muscle cells (SMCs)^52^ and a very recent one found an association between YAP and MRTF in sphingosine-1 phosphate-stimulated glioblastoma cells^53^, albeit the structural basis of these observations has not been addressed. We propose that these interactions are also mediated by the PPXY motif, suggesting that WW domain-dependent interaction between the members of the myocardin family and various Hippo effectors is a general phenomenon. However, disruption of the WW binding does not abolish TAZ/MRTF association, implying additional binding modes. Indeed, TEAD1 (a direct interactor of TAZ/YAP) can bind to the Q-rich region in the N terminus of myocardin^54^. While the isolated MRTF N terminus (as opposed to the C terminus) was insufficient to precipitate TAZ, it likely contributes to the TAZ/MRTF association, presumably via TEADs.

An important aspect of this study is the characterization of the impact of the TAZ/MRTF interaction on the nuclear transport of these proteins. This represents a novel and general regulation since myocardin, whose expression is restricted to muscle cells, is constitutively nuclear^55^, whereas the ubiquitously expressed MRTFs shuttle between the nucleus and the cytosol. Contrary to our initial assumption, MRTF does not escort TAZ into the nucleus and does not mediate F-actin-modulated regulation of TAZ traffic. This conclusion is based on our findings that the kinetics of the stimulus-induced nuclear uptake of MRTF and TAZ are different (MRTF preceding TAZ); that downregulation of MRTF augments rather than reduces nuclear entry of TAZ; that a nuclearly targeted MRTF mutant causes only modest increase in nuclear TAZ; and that targeting MRTF or TAZ to different compartments (nucleus versus cytosol) disrupts their interaction rather than relocalizing the partner. This conclusion is in agreement with observations showing that a polymerization-incompetent actin (R62D) did not impair nuclear TAZ/YAP accumulation^11^. Albeit many conditions that increase F-actin levels support TAZ/YAP nuclear entry^19^,^20^,^21^, an increase in F-actin *per se* does not seem to be sufficient. We found that strong actin polymerization by JK, which induces robust MRTF translocation, is a poor stimulus for nuclear TAZ uptake; similarly, an F-actin-stabilizing actin mutant failed to increase nuclear TAZ/YAP^11^. Instead, actomyosin-based contractility might be the critical trigger^11^,^21^, which is only a modulating factor in the case of MRTF^13^. These findings imply overlapping but distinct regulatory inputs for the two proteins. How F-actin polymerization and contractility regulate TAZ/YAP nuclear uptake remains an enigma. A recent elegant work proposes that angiomotins (AMOTs) may serve as an important link, because AMOTS can retain YAP in the cytosol, and F-actin competes with YAP for AMOT130 binding^56^.

Although the nuclear entry of MRTF and TAZ are not directly coupled, these factors profoundly influence each other's nucleocytoplasmic shuttling. The central theme is that they act as cytosolic retention factors for the partner, as deletion of one increases the cytosolic mobility and facilitates the spontaneous or stimulus-induced nuclear uptake of the other. MRTF can counteract TAZ translocation not only by direct binding but also by impacting on other cytosolic TAZ retention factors. We show that MRTF depletion results in reduced expression of 14-3-3 proteins, which are key cytosolic buffers for TAZ^57^. Moreover, we found that the expression of TAZ (but not YAP) is MRTF-dependent. These observations are in agreement with a recent genome-wide ChIP-seq analysis of putative SRF/MRTF target genes, which includes TAZ and some 14-3-3 members^24^. Finally, while our manuscript was under revision, a study (ahead of print) reported that MRTF transcriptionally regulates TAZ in breast cancer cells^58^. Together these findings suggest that MRTF is a master regulator of mechanotransduction, regulating not only the expression of its cytoskeletal targets but also the components of the Hippo pathway. Regarding the reciprocal situation, we found no evidence of TAZ-dependent regulation of MRTF expression, pointing to a hierarchical relationship. Of note, TEAD2 has been reported to contribute to myocardin expression in neurocrest-derived SMCs^59^, and microarray data suggest a weak induction of the myocardin mRNA by YAP but not TAZ^45^. It remains to be tested if YAP might regulate MRTF expression too.

We also addressed the interaction between MRTF and TAZ with regards to their transcriptional activities. In the context of the SMA promoter, which harbours the relevant cis-elements side by side, we found mutual inhibition. One arm of these effects (the inhibition of MRTF by TAZ) is analogous to the recently reported inhibition of myocardin by YAP in SMCs^52^, which has been proposed to play an important role in the switch between the synthetic (myocardin) and proliferative (YAP) states of smooth muscle, a key feature of regeneration after vascular injury. We extend this picture in several aspects. First, MRTF (unlike myocardin) is ubiquitously expressed, thus its regulation by TAZ generalizes the importance of this circuit beyond vascular remodelling, and suggests a key role in other pathologies for example, in organ fibrosis^40^,^41^,^42^,^43^ (see below). Second, it posits that WW-dependent interactions are central in the transcriptional crosstalk between TAZ/YAP and myocardin family members. DNA binding of the relevant factors seems to contribute to this inhibition, as a higher concentration of TAZ is needed to inhibit MRTF in the absence of TBE in the SMA promoter. Third, we propose that MRTF and TAZ may act antagonistically or synergistically, depending on two key factors: (1) the vicinity of the CArG boxes and TBEs; and (2) the activation state of other interacting partners (for example, Smad3) as dictated by the particular stimulus (see below). In co-expression studies we found antagonism on the SMA promoter where the CArG boxes and the TBEs are close, but synergy on isolated TBEs, presumably due to the strong transactivation domain of MRTF. Intriguingly, these cis-elements are associated in a large number of promoters^24^,^60^ suggesting that such combinatorial regulation is physiologically important. Indeed very recently a synergy was reported between MRTF and YAP on the CTGF promoter, in accordance with our findings^53^. Future studies are warranted to analyse which genes are regulated synergistically or antagonistically under various stimulatory conditions by MRTF and TAZ/YAP. The other arm of the relationship, that is, the inhibition of TAZ by MRTF is an entirely novel finding. This effect is also facilitated by the proximity of the promoter elements, as the deletion of CArG boxes vastly potentiates the effect of TAZ through TBE. A key insight generated by these studies is that TAZ confers Smad3 sensitivity to the SMA promoter. This is an important step forward as the impact of Smad3 on SMA expression has been highly controversial, with reports for both positive and negative effects (see ref. 61 for a review). In light of our new data, these apparent contradictions can be reconciled. Smad3 (ref. 25) and TAZ (this work) both inhibit MRTF, meanwhile TAZ allows Smad3 to activate the SMA promoter via SBEs; whether this effect reflects the capacity of TAZ to act as a Smad3 retention factor^36^ and/or the Smad3/TAZ complex binds with higher affinity to SBEs remains to be determined.

**Figure 10:**
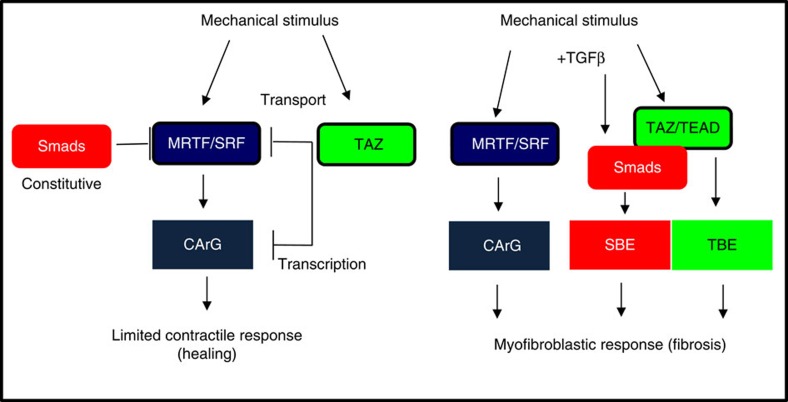
Interplay among MRTF, TAZ and Smad3 in the context-dependent regulation of the SMA promoter. TAZ as a switch between mechanical and mechanical/chemical signalling. Under resting conditions MRTF is inhibited by both TAZ and Smad3. On mechanical stimulation MRTF and TAZ translocate to the nucleus, but TAZ keeps MRTF in check by hindering its transport and inhibiting its transcriptional activity. Therefore, mechanical stimuli (increased substrate stiffness, stretch, loss of cell contacts and so on) will provoke only mitigated responses, which, although stimulate MRTF signalling and thereby cellular contractility, will not provoke substantial SMA expression and full-blown myofibroblast transition. This response may be critical for normal contraction of epithelial cells or fibroblasts during wound healing. This mechanism also ensures that Rho-activating mechanical stimuli do not automatically provoke a phenotype shift. If TGFβ is present along with mechanical stress, TAZ and Smad3 associate. This has a dual consequence. First, the complex becomes a potent activator of the SMA promoter, primarily through SBEs, that is, TAZ confers Smad3 sensitivity to the SMA promoter and second, while associating with each other, these factors dissociate from MRTF, thereby disinhibiting the activity of this critical mediator. These changes result in the expression of SMA (and an array of other MRTF-dependent and/or TAZ/Smad3-dependent proteins) and myofibroblast transition. This mechanism may play a key role in the pathogenesis of organ fibrosis. In addition to this basic scheme, MRTF also regulates TAZ expression, and our earlier studies suggest that TAZ controls Smad3 expression (not shown). Such pre-transcriptional and transcriptional crosstalk among these three central cytoskeleton- and TGFβ-regulated transcription factors ensure context-dependent integration of mechanical and chemical cues that, in turn, will determine the final response, which could be adaptive (healing) or maladaptive (fibrosis).

Our studies reveal that TAZ is a context-dependent switch between mechanical and mechanical+chemical regulation of the same gene (SMA) (Fig. 10). When contact disruption or stretch is applied alone, TAZ (and Smad3 constitutively) inhibit SMA (and SRF) expression, ensuring that isolated mechanical stimuli (for example, Rho-dependent contractility during wound healing) will not induce EMT/EMyT. When TGFβ is present during stretch, TAZ and Smad3 form a complex. This not only liberates MRTF but also assigns a dominant role for TAZ and Smad3 to drive the SMA promoter via SBEs. Thus, under these two-hit conditions the system works as a coincidence detector. This ‘Smad3/TAZ phase' may be replaced by an ‘MRTF phase' at later times when Smad3 levels decrease^25^. Smad3/TAZ-induced regulation of SMA expression may be very important in myofibroblasts, the culprit of organ fibrosis. Selective inhibition of SMA expression in myofibroblasts without altering myocardin-dependent expression in SMCs may have a large therapeutic potential. Intriguingly, previous studies support the notion that TAZ-dependent SMA regulation may be specific to myofibroblasts. An elegant study from the Owens lab^62^ revealed that TBEs (a.k.a. MCAT) contributed to SMA promoter activity in smooth muscle during development but not in adult animals. However, in adult mice TEAD-dependent reporter activation occurred on skin wounding selectively in myofibroblasts. Distinct regulation of the SMA promoter in myofibroblasts offers the possibility of selective inhibition of SMA expression, and may lead to strategies that selectively eliminate myofibroblasts during fibrosis. Such selective regulation does not contradict that MRTF is a key contributor to myofibroblast transition as well (reviewed in ref. 63). In fact, our finding that TAZ expression itself depends on MRTF underlines the importance of this master regulator both in healing and fibrogenesis.

In summary we have uncovered multilevel crosstalk between two main mechanosensitive transcription factors, which likely plays important roles in the regulation of complex (patho)physiological processes, including normal wound healing and fibrosis.

## Methods

### Reagents and antibodies

For western blot analysis, proteins were detected using antibodies obtained from various commercial sources including anti-TAZ (BD Biosciences, 560,235, 1:1,000) and anti-HA (Covance, MMS-101P, 1:1,000). Antibodies purchased from Santa Cruz Biotechnology included anti-c-Myc (sc-40, 1:1,000) anti-MRTF (sc-47282, 1:500) anti-pan-14-3-3 (sc-629, 1:1,000), anti-GAPDH (sc-47724, 1:20,000), anti-CTGF (sc-14939, 1:1,000) and anti-Cyr61(sc-8561, 1:1,000). Cell Signaling Technology was the source for anti-Smad3 (#9513, 1:1,000), anti-YAP/TAZ (#8418, 1:1,000) and anti-SRF (#5147, 1:1,000), and Sigma-Aldrich for anti-FLAG/M2 (F1804, 1:1,000) and anti-SMA (AF228; 1:5,000). Anti-histone was from EMD Millipore (MAB052, 1:500) and anti-Filamin A was from Abcam (ab51217, 1:500). Anti-BSAC antibody was a gift from H. Nakano and was described previously^64^. Normal goat, rabbit and mouse IgG were from Santa Cruz Biotechnology (sc-2028, sc-2027 and sc-2025, respectively). Jackson ImmunoResearch Laboratories was the source for all horseradish peroxidase-conjugated secondary antibodies (1:5,000). TGFβ was purchased from R&D Systems and JK from EMD.

### Cell culture

LLC-PK1 cells were cultured in low-glucose DMEM (Life Technologies), supplemented with 10% FBS. Cells were incubated under serum-free conditions for 3 h before treatments or experimental procedures. Cell contact disassembly was obtained by thoroughly washing cultures in PBS and placing them in nominally calcium chloride-free DMEM (LCM). Where indicated, cells were treated with 5–10 ng ml^−1^ TGFβ.

### Expression plasmids and siRNA transfection

HA-tagged WT and mutant (S89A) TAZ constructs were gifts from Kunliang Guan (plasmids 32,839 and 32,840; Addgene, Cambridge, MA). Full-length FLAG, Myc- or HA-tagged MRTF constructs were generated as described previously^25^. Standard PCR techniques were used to create the N- or C-terminal MRTF and to clone both WT MRTF and TAZ into the pEGFP expression vector. Single or double point mutations were created by standard site-directed mutagenesis using a high-fidelity proof-reading polymerase (iProof; BioRad or Phusion; Thermo Scientific). All new constructs generated were verified by sequencing. Transfection with expression vectors was performed using X-Treme Gene 9 (Roche Applied Science); Lipofectamine 2000 (Invitrogen) or jetPrime (PolyPlus Transfection SA).

Porcine-specific siRNA used in knockdown experiments were directed against the following sequences : TAZ (#1) 5′-GGAAGAAGATCCTGCCTGA-3′ or TAZ (#2) 5′-CAAGAACATACACCTACGGTTGT-3′; YAP 5′-TCAAAGCGCTCCAGTGAAA-3′; MRTF A 5′-AACCAAGGAGCUGAAGCCAAA-3′; and MRTF B 5′-AACGACAAACACCGTAGCAAA-3′ (ref. 65). Key findings obtained on TAZ downregulation were confirmed by using both TAZ #1 and #2 siRNA. Equal molar concentrations of MRTF A and MRTF B siRNA were used in all experiments. Oligonucleotides were synthesized and purchased from Thermo Scientific or Sigma-Aldrich. The concentration and duration required to achieve optimal silencing were validated by western blotting and quantitative PCR (qPCR). NR control siRNA was obtained from Applied Biosystems and used under the same experimental conditions. Transfections with siRNA alone were performed using LipofectamineRNAiMAX (Invitrogen) and co-transfections with cDNA and siRNA were carried out using jetPrime.

### Luciferase reporter assay

The SBE4-Luc and the p765-SMA-Luc (WT) reporter construct, including the subsequent mutations in both the CArG boxes and the SBE sites, was previously described in detail^25^. Mutation of the two putative TEAD-binding elements (also called MCAT elements) in the WT SMA-Luc promoter was performed using standard site-directed mutagenesis techniques. The TBE2mut promoter was generated first (G^−318^/T; G^−317^/T) and then used as the template to generate the TBE1/2mut promoter (G^−182^/T; G^−181^/T). The CArG A/B_TBE1/2 construct was generated as described above using the CArG A/B mutated SMA-Luc as the template. The WT TEAD and inactive TEAD luciferase reporter constructs were gifts (Dr L. Attisano) and have been previously described^66^. The CTGF promoter (−805) luciferase construct was a gift from Dr A. Leask^67^. Cells were transfected with reporter constructs together with the normalizing plasmid pRL-TK (Promega) and the indicated expression plasmids and/or siRNA. Supplementation of the transfection mixture with empty vector, when required, ensured that the total amount of DNA transfected remained constant in all samples. Renilla luciferase and firefly luciferase activities in cell lysates were measured using a reporter assay system (Dual Luciferase; Promega) in a luminometer (Lumat 9507; Berthold). Transfections and measurements were performed in triplicates for each experiment and experiments were repeated three times. Results are expressed as fold changes compared with the mean firefly/renilla ratio of the control taken as a unit.

### Immunoprecipitation and western blotting

To examine the interaction between endogenous MRTF, TAZ and Smad3, or the indicated, transiently transfected, tagged proteins, LLC-PK1 cells were collected from 10-cm dishes or duplicate wells from a 6-cm BioFlex plate following any described treatment. The lysis buffer (30 mM HEPES (pH 7.4), 100 mM NaCl, 1 mM EGTA, 20 mM sodium fluoride and 1% Triton X-100) was supplemented with 1 mM PMSF, 1 mM sodium vanadate and Complete Mini Protease Inhibitor (Roche). Lysates were spun at 12,000 r.p.m. for 5 min to remove cell debris and analysed for protein content (BCA Protein Assay; Pierce Biotechnology). Precleared supernatants were incubated with the precipitating antibody or a control IgG and then with aliquots of Protein A/G UltraLink Resin (Thermo Scientific). Bound proteins were eluted from the washed beads and subjected to SDS–PAGE followed by western blot analysis. Aliquots of each input were run in parallel to monitor expression levels. Immunodetection was performed using either ECL or ECL Plus reagents (GE Healthcare, Life Sciences) and densitometric analysis was performed using a GS800 densitometer and Quantity One software (BioRad). Cell lysates obtained following specified treatments or gene silencing were also processed by immunoblotting with the indicated antibodies. All experiments were performed a minimum of three times and representative immunoblots are shown.

### Chromatin immunoprecipitation assays

Assays were carried out on LLC-PK1 cells transfected with NR or TAZ siRNA, followed by treatment with or without LCM or on non-transfected monolayers treated with LCM in the presence or absence of TGFβ. ChIP was performed essentially following the manufacturer's protocol (Millipore). After immunoprecipitation with 2.0 μg anti-MRTF, anti-TAZ antibody or the appropriate control IgG, qPCR was performed on the recovered DNA using primers encompassing the CArG box elements within the pig SMA promoter.

### Immunofluorescence microscopy

Cells grown on glass coverslips or BioFlex membranes were transfected and/or treated as indicated in the figures. The samples were then fixed with 4% paraformaldehyde, at which point the silicone membrane from the BioFlex plate was excised and cut into sections to allow multiple staining. Following permeabilization with 0.1% Triton X-100 and blocking with BSA, the cells were incubated with the indicated primary antibodies as follows: Flag/M2 (Sigma, F1804,1:50); TAZ (Santa Cruz, sc-17130, 1:100); MRTF (sc-47282, 1:50); c-myc (Santa Cruz, sc-40, 1:50); HA (Covance, MMS-101P, 1:300); and BSAC (1:100). After washing the appropriate fluorescently conjugated secondary antibody (Alexa 488 or 555; Invitrogen, 1:1,000) was applied. 4,6-diamidino-2-phenylindole (Lonza) was used to counterstain nuclei. Coverslips were mounted on slides using fluorescent mounting medium (Dako). Images were captured using an Olympus IX81 microscope coupled to an Evolution QEi Monochrome camera using MetaMorph Premiere software. Quantification of nuclear localization was evaluated by examining at least 10 randomly selected fields per each condition in a minimum of three independent experiments. The scale bar is 10 μm in each figure. All image processing was done according to the Journal's guidelines.

### Confocal microscopy and FRAP experiments

LLC-PK1 cells plated on 25-mm glass coverslips were co-transfected with either GFP MRTF along with NR or TAZ-specific siRNA or GFP TAZ and NR or MRTF-specific siRNA. Experiments were performed 48 h later. Cells were placed in a TC-L-10 live incubation device and live images were taken using a WaveFX spinning-disk microscopy system (Quorum Technologies, Guelph, Canada) equipped with ORCA-Flash4.0 digital camera with Gen II sCMOS image sensor and an ILas2 FRAP module, driven by the Metamorph software. Three images at 3-s intervals (pre-bleach) were acquired before bleaching the cytosolic region of interest with a 488-nm laser (bleach). In all, 20 post-bleach images were collected every 360 ms followed by further 16 images every 3 s for a total of 40 images. Data were collected from an average of at least 10 cells for each condition from the bleached region of interest, a non-bleached region within the same cell and a non-bleached control region outside of the cell of interest. Data were corrected point by point for spontaneous photobleaching (which was <2% in the investigated time frame). Normalization of raw recovery curves and curve fitting were performed using easyFRAP; a stand-alone analysis tool^68^.

### Mechanical cell stretch

Cells were plated onto six-well plates with untreated flexible bottoms (BioFlex culture plates) and subjected to a 1 or 6 h stretch regimen by software-controlled vacuum applied to a loading station housed in a humidified 5% CO_2_ incubator at 37 °C (Flexcell 5000). Each cycle consisted of 0.5 s of stretch (10%) and 0.5 s of relaxation for a total of 60 cycles per min. The replicate control plates consisted of cells grown on the same flexible surface but not subjected to mechanical stretch.

### Nuclear extraction

LLC-PK1 cells were treated as indicated and nuclear extracts were prepared using the NE-PER Nuclear Extraction kit (Thermo Scientific). Total and nuclear extracts were analyzed by Western blotting. Equal loading of nuclear proteins were verified using an anti-histone antibody.

### Quantitative PCR

LLC-PK1 cells were transfected or treated as indicated and total RNA was extracted using the RNeasy Kit (Qiagen). Following cDNA synthesis using iScript reverse transcriptase (BioRad), SYBR green-based real time PCR was performed to evaluate gene expression of TAZ using GAPDH as the reference standard. Primer pairs were as follows: *TAZ* 5′-GATGAGATGGACACAGGAGAAA-3′ and 5′-CCCGGAAGACAGTCAAGAAA-3′ ; *GAPDH* 5′-GCAAAGTGGACATGGTCGCCATCA-3′ and 5′-AGCTTCCCATTCTCAGCCTTGACT-3′.

Each sample was analysed in triplicate and experiments were performed three times.

All qPCR experiments were performed using an IQ cycler (BioRad).

### Statistics

Data are presented as blots or images from at least three similar experiments or as the means±s.e.m. for the number of experiments indicated (*n*). Statistical significance was determined by Student's *t*-test or one-way analysis of variance (Tukeyposthoc testing) as appropriate using Prism software (version 6.02 ,GraphPad Software, Inc.). *P*<0.05 was accepted as significant, *, ** and *** correspond to *P*<0.05, <0.01 and <0.001, respectively.

### Data availability

All relevant data are available from the authors.

## Additional information

**How to cite this article:** Speight, P. *et al.* Context-dependent switch in chemo/mechanotransduction via multilevel crosstalk among cytoskeleton-regulated MRTF and TAZ and TGFβ-regulated Smad3. *Nat. Commun.* 7:11642 doi: 10.1038/ncomms11642 (2016).

## Supplementary Material

Supplementary Information Supplementary Figures 1-4
