# Supplementary material for: Context-dependent switch in chemo/mechanotransduction via multilevel crosstalk among cytoskeleton-regulated MRTF and TAZ and TGFβ-regulated Smad3
Source: Nat Commun. 2016 May 18;7:11642. doi: 10.1038/ncomms11642 (PMC4873981; doi:10.1038/ncomms11642)
Supplement: Supplementary Information — Supplementary Figures 1-4 [file ncomms11642-s1.pdf]

a

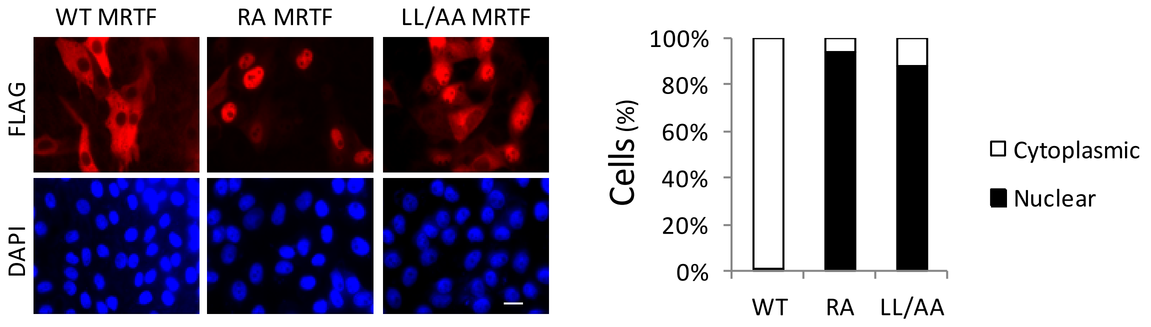

b

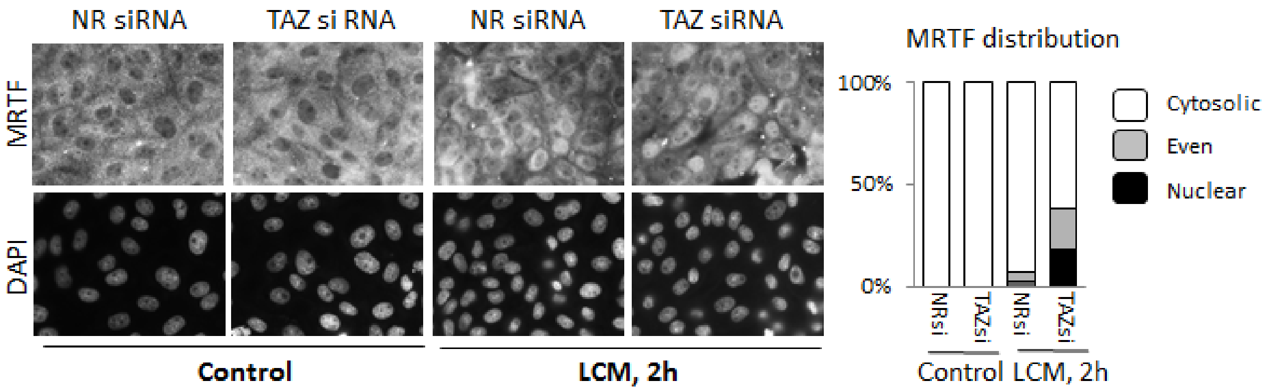

Supplementary Figure 1. Intracellular distribution of WT and mutant MRTF under various conditions. a) Cells transfected with FLAG-tagged WT, RA, or LL/AA MRTF were stained for the FLAG epitope. Nuclei were stained with DAPI. The percentage of cells showing cytosolic or nuclear MRTF localization was quantified by immunofluorescence microscopy. An average of 150 cells/condition were analyzed for n=3 experiments. **b)** Cells were transfected with non-related (NR) siRNA or a TAZ siRNA (#2), Twenty-four h later cells were serum-deprived and then exposed to serum-free control or low calcium medium (LCM) for 2 h. Cell were then fixed and stained for MRTF. Nuclei were visualized by DAPI. Intracellular distribution of MRTF was determined in 200-300 cells/condition in 2 experiments. Note that >4-fold higher number of cells exhibited nuclear MRTF accumulation in TAZ-silenced than in control cells at this point, in agreement with the results obtained with TAZ siRNA (#1), as shown in Figure 3b. Scale bar, 10μm.

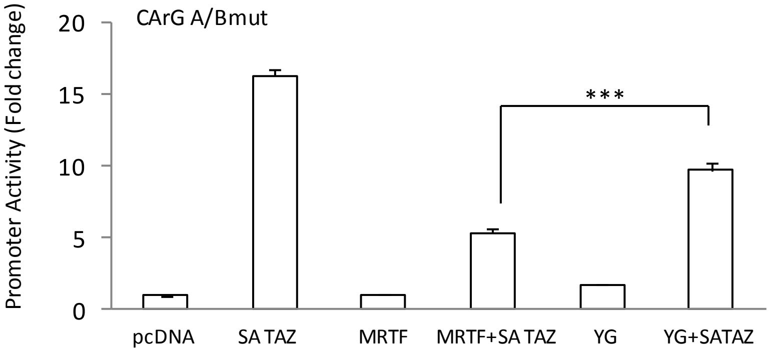

Supplementary Figure 2. The PPXY motif in MRTF contributes to the inhibitory action of MRTF on TAZ, observed on the CArGmut SMA promoter.
The CArG A/B mutant SMA promoter was cotransfected with control plasmid (pcDNA), SA TAZ, WT MRTF (MRTF), YG MRTF (YG), or combinations as shown, and luciferase assays were performed as described in Fig. 6b.
Error bars represent ± s.e.m. for n=3 independent experiments. ***P <0.001 from pairwise t-test.

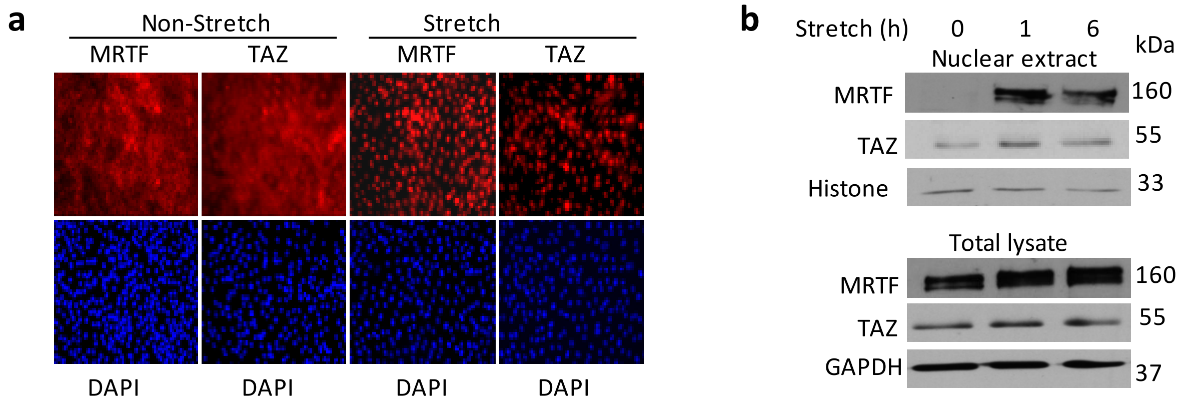

Supplementary Figure 3. Cyclic stretch translocates both MRTF and TAZ into the nucleus.
(a) LLC-PK1 cells gown to confluence on silicone bottomed Flexcell plates exposed to cyclic stretch (10% at 1 Hz for 1 or 6 h). Cells were then either fixed and processed for immunostaining for TAZ and MRTF or **(b)** processed for isolation of nuclear fractions and whole cell fractions, as described in the Methods, followed by Western blotting for MRTF, TAZ and nuclear (histones) or cytosolic/whole cell (GAPDH) markers.

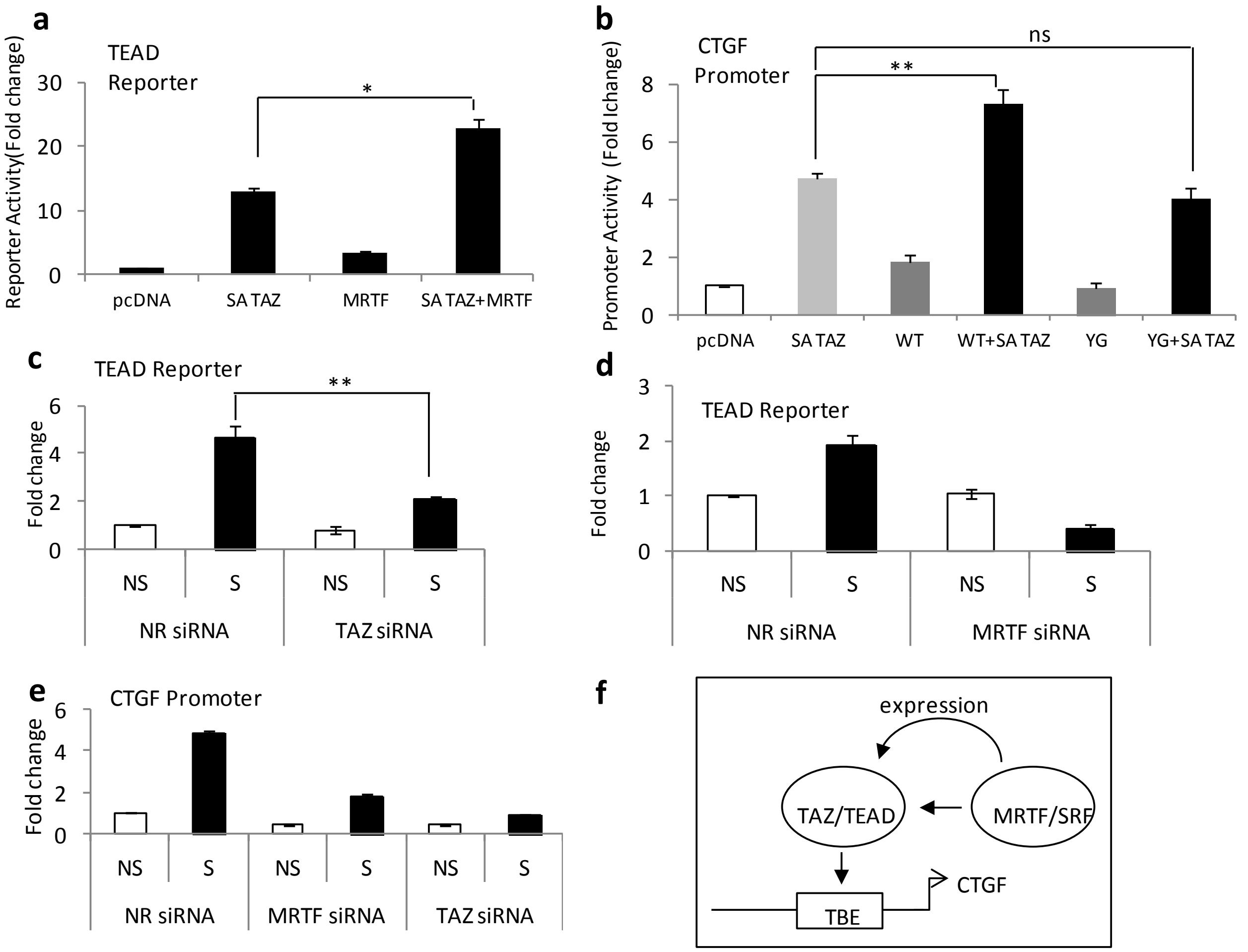

Supplementary Figure 4. TAZ and MRTF act synergistically on TEAD elements not adjacent to a CArG box.

In the absence of TGFβ, MRTF and TAZ are antagonistic on the SMA promoter. To test if this interaction is different on TBEs which are not located in the vicinity of CArG boxes, **(a)** cells were cotransfected with a TEAD reporter luciferase construct (containing 8 tandem TBEs) and either an empty vector (pcDNA) or SA TAZ, MRTF or both. Note that TAZ had a strong stimulatory effect, which was significantly enhanced by MRTF. n=3. **(b)** The effect of MRTF (WT) and YG MRTF (YG) on the connective tissue growth factor (CTGF) promoter. MRTF and YG MRTF were cotransfected with or without SA TAZ and a luciferase reporter harboring an 805 bp segment of the CTGF promoter, which is a prominent TAZ target. This region of the promoter contains TEAD elements but no CArG box[65]. SA TAZ substantially stimulated this construct, while MRTF had a modest effect itself but significantly enhanced that of TAZ. This stimulatory/ potentiating effect was lost when YG MRTF was used. These findings imply that MRTF and TAZ synergize on TBEs in a WW motif-dependent manner. n=3. **(c)** and **(d)** Silencing TAZ or MRTF abrogates the stretch-induced activation of the TEAD reporter. Cells were cotransfected with NR or TAZ siRNA **(c)** or MRTF siRNA **(d)** along with the TEAD reporter. Twenty-four hours later cells were left unperturbed (non-stretch, NS) or subjected to cyclic stretch (S, 10%, 1 Hz for 1 h) and after 24 h processed for luciferase assays. Note that stretch caused significant activation of the reporter, which was strongly inhibited by TAZ siRNA, indicating the contribution of TAZ to transcriptional responses in the mechanically challenged monolayer. n=4. Downregulation of MRTF abrogated the activation of the reporter, an effect attributable primarily to the MRTF-dependence of TAZ expression. n=4. **(e)** The impact of MRTF and TAZ on the stretch-induced activation of the CTGF promoter. Cells were transfected with NR, MRTF or TAZ siRNA along with the CTGF promoter construct, and luciferase activity was measured after the indicated treatment. Note that stretch potently activated the CTGF promoter, which was mitigated by MRTF siRNA and strongly suppressed by TAZ siRNA. n=3. Thus, TAZ and MRTF are required for the efficient transmission of mechanical stimuli through TBEs. **(f)** Schematic representation of the synergy between TAZ and MRTF on TBEs suggesting a potential mechanism whereby MRTF, in complex with TAZ, lends its strong transactivation domain to TAZ-driven promoters. Additionally, MRTF enhances TAZ expression (curved arrow). For **(a-e)**, error bars denote ±s.e.m. *P <0.05, **P <0.01, ***P <0.001 and ns = not significant.
